# Serum and Vitreous Concentrations of Omentin-1 in Diabetic Retinopathy

**DOI:** 10.1155/2015/754312

**Published:** 2015-09-03

**Authors:** Wencui Wan, Qiuming Li, Fengyan Zhang, Guangying Zheng, Yong Lv, Guangming Wan, Xuemin Jin

**Affiliations:** Department of Ophthalmology, The First Affiliated Hospital of Zhengzhou University, Zhengzhou, China

## Abstract

*Objective*. Omentin, a new discovered adipokine, is implicated to inhibit inflammation. Inflammation is one important mechanism of diabetic retinopathy (DR). The purpose of this work was to evaluate serum and vitreous concentrations of omentin-1 in patients with diabetic retinopathy (DR). *Methods*. This study enrolled 204 diabetic patients (60 without DR, 49 with NPDR, and 95 with PDR) and 65 control subjects. *Results*. Serum and vitreous omentin-1 levels in PDR patients were markedly decreased compared with those in the other three groups. NPDR patients showed reduced vitreous omentin-1 compared with patients without DR. In addition, control subjects had significantly higher levels of serum and vitreous omentin-1 compared with diabetic patients without DR, NPDR patients, and PDR patients. In addition, serum/vitreous omentin-1 ratio was positively correlated with the development and severity of DR. *Conclusion*. Serum and vitreous omentin-1 levels, as well as serum/vitreous omentin-1 ratio, are correlated with the presence and severity of DR.

## 1. Introduction

Diabetic retinopathy (DR), the most serious diabetic complication, is estimated to account for 4.8% of global blindness [1]. Although major research has been concentrated on the elucidation of its pathogenesis, the clear mechanism of DR is still unknown. Within the factors involved in the mechanism of DR, inflammation is paid more attention during the past decade [2]. A multitude of inflammatory mediators have been demonstrated to be involved in the mechanism of diabetic retinopathy [3].

Omentin-1, a novel visceral fat depot-specific secretary protein, is produced/synthesized by the visceral stromal vascular cells [4]. Omentin-1 could increase insulin-stimulated glucose uptake and Akt phosphorylation in human adipocytes [4]. Decreased omentin-1 concentrations were found in patients with insulin resistance (IR) [5] and diabetes mellitus [6]. Recently, omentin-1 was demonstrated to play an anti-inflammatory role in vascular smooth cells [7]. Inflammation is correlated with the development of DR. Therefore, omentin-1 is hypothesized to play a role in DR development.

To our knowledge, the role of omentin-1 in the pathogenesis of DR has not been examined previously. With this background in mind, we designed this study to investigate the association of serum and vitreous omentin-1 levels with the presence of DR.

## 2. Materials and Methods

### 2.1. Patients

A consecutive population of 204 patients who were diagnosed with type 2 diabetes mellitus (T2DM) and underwent vitrectomy were divided into T2DM patients without DR group (*n* = 60), non-PDR (NPDR) group (*n* = 49), and PDR group (*n* = 95). Subjects were excluded if they had chronic systemic disease, cancers, ocular disorders, previous intraocular surgery or laser treatment, photocoagulation, and intravitreal hemorrhages obvious vitreal hemorrhage within the previous 3 months. Sixty-five patients who underwent vitrectomy for retinal detachment with no systemic disease were recruited in the control group. The control group matched with T2DM patients in age, gender, and BMI.

The study was planned according to the ethics guidelines of the Helsinki Declaration and was approved by the Institutional Research Ethics Board of our university. All patients gave written informed consent regarding participation in this study.

### 2.2. Measurements

Serum was obtained from blood samples by centrifugation and was stored at −80°C until analysis. Vitrectomy samples (approximately 0.3 mL) were got using a standardized vitrectomy technique with manual suction before opening the infusion set. The serum and vitreous samples were analyzed for omentin-1 using commercially available enzyme-linked immunosorbent assay (Cusabio Biotech Corporation, USA).

### 2.3. Statistical Analysis

Data are presented as means ± SD or median (interquartile range). Kolmogorov-Smirnov test was utilized to determine data normality. The differences of characteristics between patients with PDR and NPDR, diabetic patients without DR, and control subjects were compared using Chi-square tests, one-way ANOVA, or Kruskal-Wallis test. Statistical analysis was carried out using SPSS version 13.0 software program (SPSS Inc., Chicago, Illinois). *P* values less than 0.05 were considered to be statistically significant.

## 3. Results

### 3.1. Baseline Clinical Characteristics

As shown in Table 1, T2DM patients had elevated levels of systolic blood pressure (SBP), diastolic blood pressure (DBP), HbA1c, fasting plasma glucose (FPG), and triglycerides (TG) compared to the controls. Subjects with PDR showed markedly decreased HDL-C levels compared with the other three groups.

#### 3.1.1. Serum and Vitreous Omentin-1 Levels

Serum and vitreous omentin-1 levels in the four groups are shown in Table 2. Serum and vitreous omentin-1 levels in PDR patients were both significantly decreased compared with those in the other three groups. There were markedly higher levels of serum and vitreous omentin-1 in the control group compared with the other three groups. Furthermore, NPDR patients showed reduced vitreous omentin-1 levels compared with T2DM patients without DR. However, there were no significant differences in serum omentin-1 levels between NPDR patients and T2DM patients without DR.

#### 3.1.2. Serum/Vitreous Omentin-1 Ratio

Serum/vitreous omentin-1 ratio is presented in Table 2. PDR patients showed significantly elevated serum/vitreous omentin-1 ratio compared with the other three groups. Serum/vitreous omentin-1 ratio was marked increased in NPDR patients compared with the controls and T2DM patients without DR. However, no significant differences of serum/vitreous omentin-1 ratio were found between the controls and T2DM patients without DR.

## 4. Discussion

Omentin-1 is a recently discovered adipokine which is preferentially produced by visceral adipose tissue. Omentin-1 treatment could enhance insulin-stimulated glucose uptake in human adipocytes. In addition, omentin increased Akt phosphorylation in human adipocytes [4]. Serum omentin-1 levels were lower in the impaired glucose regulation group than in the normal glucose tolerance (NGT) group [6]. Plasma omentin-1 levels were lower in patients with T2DM than in the controls [8]. Furthermore, decreased plasma omentin-1 levels were reported in type 1 diabetes mellitus [9]. Omentin-1 levels were significantly lower in nonobese gestational diabetes mellitus (GDM) women compared to nonobese NGT women [10]. All these findings suggest that omentin-1 may have a protective effect in the pathogenesis of diabetes.

Omentin-1 is associated with the presence of diabetic macrovascular complication. Exposure of cardiomyocytes to conditioned media derived from epicardial adipose tissue from patients with T2DM induced contractile dysfunction and insulin resistance, which was prevented by the addition of recombinant omentin [11]. Yoo et al. reported that T2DM subjects with higher baseline serum omentin-1 levels tended to have a reduced arterial stiffness after one year [12]. In addition, serum omentin-1 levels were significantly decreased in T2DM patients with carotid plaque compared to those without carotid plaque [13].

Omentin-1 is also suggested to be correlated with diabetic microvascular complication. Plasma levels of omentin were found to be markedly higher in end-stage renal disease (ESRD) patients [14]. In another study, omentin-1 levels in patients with nondiabetic chronic kidney disease were found to be higher than the control group [15]. In addition, serum omentin-1 levels in CKD patients were significantly lower compared to the healthy controls. Further analysis revealed that decreased omentin-1 in chronic kidney disease (CKD) patients was due to the reduced omentin-1 levels in the diabetic subgroup [16]. This indicates that serum omentin-1 is involved in the mechanism of diabetic nephropathy. DR is another important microvascular complication of diabetes. The present study demonstrated that serum and vitreous omentin-1 levels in PDR patients were both significantly elevated compared with diabetic patients without DR, NPDR patients, and the controls.

Vitreous omentin-1 concentrations are lower than serum omentin-1 concentrations. This indicates that vitreous omentin-1 may be caused by bleeding from the vascular system of the eyes. This hypothesis could explain the similar reduction of plasma and vitreous omentin-1 concentrations. Omentin-1 in eyes may protect the eyes against DR development. And our results showed that serum/vitreous omentin-1 ratio was positively correlated with the presence and severity of DR. This indicates that serum/vitreous omentin-1 ratio may be utilized to predict or assess the development and progression of DR.

Angiogenesis is a key mechanism of DR. Omentin-1 could significantly decrease vascular endothelial growth factor (VEGF) induced endothelial cell migration and angiogenesis in human microvascular endothelial cells [17]. This suggests that omentin-1 may serve as antiangiogenic mediator and play an important protective role in the development of DR through the antiangiogenic effects.

Omentin-1, one newly discovered adipokine, is shown to be an anti-inflammatory mediator. Omentin was found to inhibit tumor necrosis factor- (TNF-) induced vascular inflammation in human endothelial cells [18] and vascular smooth muscle cells [19]. Serum omentin-1 was reported to be inversely associated with inflammatory cytokines such as TNF-*α*, interleukin-6 (IL-6), and C-reactive protein [20, 21]. These results indicate the anti-inflammatory role of omentin-1. Inflammation has been suggested as a potential mechanism for DR. Omentin-1 may be involved in the mechanism of DR via the inhibitory role of inflammatory pathway.

In short, serum and vitreous omentin-1 levels, as well as serum/vitreous omentin-1 ratio, are correlated with the presence and severity of DR.

## Figures and Tables

**Table 1:** Various characteristics of diabetic patients and controls.

	Controls	Diabetic patients			*P*
		Without DR	NPDR	PDR	
*n*	65	60	49	95	
Age (years)	57.85 ± 6.90	58.67 ± 9.00	58.16 ± 12.66	57.32 ± 10.96	0.872
Gender (M/F)	32/33	25/35	23/26	45/50	0.852
BMI (Kg/m^2^)	23.77 ± 1.59	23.83 ± 3.00	24.32 ± 3.94	24.41 ± 3.22	0.468
SBP (mmHg)	124.23 ± 11.45	141.75 ± 21.74^a^	140.51 ± 21.44^a^	140.05 ± 23.44^a^	<0.001
DBP (mmHg)	83.11 ± 7.63	89.25 ± 9.69^a^	87.37 ± 13.48^a^	87.11 ± 12.20^a^	0.016
HbA1c (%)	5.03 ± 0.71	7.76 ± 0.95^a^	7.82 ± 1.03^a^	7.82 ± 1.30^a^	<0.001
FPG (mmol/L)	5.11 ± 0.40	8.29 ± 1.86^a^	8.59 ± 1.50^a^	8.10 ± 1.66^a^	<0.001
TC (mmol/L)	5.19 ± 0.89	5.38 ± 1.40	5.33 ± 1.10	5.33 ± 1.28	0.822
TG (mmol/L)	1.17 ± 0.31	1.81 ± 0.58^a^	1.85 ± 0.55^a^	2.03 ± 0.62^a^	0.002
LDL-C (mmol/L)	3.35 ± 0.73	3.39 ± 1.06	3.36 ± 0.92	3.52 ± 1.05	0.643
HDL-C (mmol/L)	1.45 ± 0.27	1.42 ± 0.40	1.43 ± 0.33	1.26 ± 0.30^abc^	0.001

^a^ *P* < 0.05 versus control; ^b^ *P* < 0.05 versus diabetic patients without DR; ^c^ *P* < 0.05 versus NPDR patients.

**Table 2:** Serum and vitreous omentin-1 levels in controls, diabetic patients without DR, NPDR patients, and PDR patients.

Omentin-1 (ng/mL)	Controls (*n* = 65)	Without DR (*n* = 60)	NPDR (*n* = 49)	PDR (*n* = 95)	*P* value
Serum	208.31 (164.20–251.20)^bc^	184.41 (142.50–206.14)^a^	166.97 (132.96–199.21)^a^	139.96 (119.28–157.87)^abc^	<0.001
Vitreous	96.00 (75.24–112.64)^bc^	81.46 (67.84–96.39)^ac^	64.28 (53.08–74.45)^ab^	50.36 (39.91–57.73)^abc^	<0.001
Serum/vitreous ratio	1.99 (1.72–2.63)^c^	2.17 (1.82–2.57)^c^	2.72 (2.08–3.37)^ab^	2.88 (2.31–3.48)^abc^	<0.001

^a^ *P* < 0.05 versus control; ^b^ *P* < 0.05 versus diabetic patients without DR; ^c^ *P* < 0.05 versus NPDR patients.
